# Searching for the True Diet of Marine Predators: Incorporating Bayesian Priors into Stable Isotope Mixing Models

**DOI:** 10.1371/journal.pone.0092665

**Published:** 2014-03-25

**Authors:** André Chiaradia, Manuela G. Forero, Julie C. McInnes, Francisco Ramírez

**Affiliations:** 1 Department of Research, Phillip Island Nature Parks, Cowes, Victoria, Australia; 2 Department of Conservation Biology, Applied Marine Ecology Group, Estación Biológica de Doñana, Sevilla, Andalucía, Spain; Institut Pluridisciplinaire Hubert Curien, France

## Abstract

Reconstructing the diet of top marine predators is of great significance in several key areas of applied ecology, requiring accurate estimation of their true diet. However, from conventional stomach content analysis to recent stable isotope and DNA analyses, no one method is bias or error free. Here, we evaluated the accuracy of recent methods to estimate the actual proportion of a controlled diet fed to a top-predator seabird, the Little penguin (*Eudyptula minor*). We combined published DNA data of penguins scats with blood plasma δ^15^N and δ^13^C values to reconstruct the diet of individual penguins fed experimentally. Mismatch between controlled (true) ingested diet and dietary estimates obtained through the separately use of stable isotope and DNA data suggested some degree of differences in prey assimilation (stable isotope) and digestion rates (DNA analysis). In contrast, combined posterior isotope mixing model with DNA Bayesian priors provided the closest match to the true diet. We provided the first evidence suggesting that the combined use of these complementary techniques may provide better estimates of the actual diet of top marine predators- a powerful tool in applied ecology in the search for the true consumed diet.

## Introduction

Searching for the true diet consumed by meso-top marine predators is of great significance in foraging ecology research. Large marine vertebrates are major consumers in marine ecosystems [e.g. seabirds, 1,2]. Their diet can offer insights into the fluctuations of fish stocks and overall marine ecosystem variability [3]–[7]. Data on diet and changes in their trophic habits can also provide information on dramatic changes in prey composition [8] or in oceanographic conditions [9] and these data are crucial building blocks in ecosystem models [10], [11]. However, the use of large marine vertebrates as indicator of changes in the marine food webs depends on an accurate evaluation of their diet [12]–[14].

Despite the existence of different methods to assess diet composition of marine animals, from conventional stomach content analysis to more recent stable isotope and DNA analyses, no method is bias or error free [12], [15]–[19]. An early study suggested that at least one method should be used to measure the numerical abundance and another to quantify the volume of the same diet samples [16]. Since then, several studies have combined two independent methods of sampling and several methods of data analysis to deal with biases. Recently, combined methods from independent sources, like stomach contents and stable isotope analysis have been used to better estimate diet composition [20]–[22] or to validate DNA analysis technique [23].

But these methods also have their limitations. While DNA analysis provides a comprehensive list of prey species, it only provides information on prey consumed within one foraging trip [12]. Prey may not be detected if DNA have been degraded during storage or prey DNA is absent in the faeces because the animal had not fed recently [23]. In addition, the reliability of quantitative data recovered from DNA-based studies is only beginning to be examined [14], [17]. Stable isotopes integrate the diet over a relatively longer period (depending on tissue analysed) but the quantification of prey at a species level is difficult to estimate when prey isotopic signatures are not distinctive [24]. While these two techniques seem to be complementary by providing diet assimilated (stable isotope) and diet digested (DNA analysis); no study to our knowledge has combined stable isotope and DNA analyses in dietary reconstructions. Further, mixing models using a Bayesian approach is providing the ability to integrate stable isotopes with other data sources in one single *a posteriori* model by including supplementary information based on previous dietary knowledge (priors) to further refine dietary analysis [25]–[28].

In this paper, we evaluated which recent method of dietary analysis most accurately reflected the actual diet of experimentally fed marine predator, little penguins (*Eudyptula minor*, Forster 1781). We estimated prey contributions using 1) mixing models based on stable isotope values (δ^15^N, δ^13^C) and 2) Bayesian mixing models incorporating priors, derived from DNA analysis of penguin scats from same individuals in the same feeding trials [29]. Before running the mixing models, we performed a separate feeding experiment to determine the trophic enrichment factor of consumer tissues relative to their diet [30] given that small differences in the enrichment factor can completely alter the output of model [31].

## Methods

A captive feeding experiment was performed between December 2009 and January 2010 at the Phillip Island Nature Parks wildlife rehabilitation centre to estimate the specific trophic enrichment factor for red cells and plasma of little penguins. Captive adult penguins in this rehabilitation centre were part of a routine rehabilitation program. During the period of their rehabilitation (mean 35 days, range from 8 to 71 days), penguins were fed a diet of 100% whole defrosted sardines (,*Sardinops sagax* Jenyns 1842). Fish muscle samples were collected from the same batch of sardines fed to penguins (n = 20). Blood samples from birds were collected when they were healthy, just before being released in the wild. Out of 19 blood samples, 14 plasma samples had enough material for lab analysis. Nine red cell samples, only from penguins >30 days in captivity in order to account for 28-days turnover [32], were used to calculate the trophic enrichment factor.

In a separate experiment in January 2008, a different group of penguins (n = 30) were 8 to 9 week old fledglings when they were removed from nesting burrows located in high-risk nest trampling areas of Phillip Island, Australia (38°15′S, 145°30′E) as part of an ongoing translocation program. At the Phillip Island Nature Parks wildlife rehabilitation centre, fledglings were kept in captivity for about one week before being released to the wild at a protected nesting site. During an initial acclimatization of three days, the birds were fed a diet of 100% whole defrosted sardines. For the final four days of captivity, the penguins were fed a ‘fish-shake diet’ (a constant mass of blended fish tissue; 100 g twice daily) and then fed until satiated with a portion of whole defrosted sardines (46% of total daily food). The fish-shake diet consisted of 45% tuna (*Scombrinae sp*), 35% tommy ruff (,*Arripis georgianus* Valenciennes 1831), and 20% whiting (,*Sillago flindersi* McKay 1985) by mass. Approximately 80 μl of blood per penguin was collected on the fifth day for stable isotope analysis. Fish muscles of all four species were sampled for stable isotope analysis as well. During this experiment, scats of penguins were also collected as part of another study that used high-throughput sequencing to characterize the prey DNA recovered from these samples, published elsewhere [29].

All blood samples were centrifuged to obtain plasma and red cells immediately after sampling. Blood and muscle samples were frozen at −18C for later analysis.

Before stable isotope analyses, all samples were freeze-dried and powdered. Lipids from muscle and plasma samples were extracted prior to analysis [33]. Stable-carbon and nitrogen isotope assays were performed on 1 mg of homogenised sample by loading into tin cups. Isotopic analyses were performed using a continuous flow isotope-ratio mass spectrometry system (Thermo Electron) consisting of a Flash HT Plus elemental analyser interfaced with a Delta V Advantage mass spectrometer. Stable isotope ratios are expressed in the standard δ-notation (‰) relative to Vienna Pee Dee Belemnite (δ^13^C) and atmospheric N_2_ (δ^15^N). Based on replicate measurements on the within-run standards LIE-BB (baleen; mean ± SD = −18.58±0.06‰ and 9.95±0.02 for δ13C and δ15N), LIE-CV (cow horn, −22.19±0.09‰ and 10.25±0.1) and LIE-PA (feather, −15.77±0.08‰ and 16.55±0.05), as previously calibrated using international standards IAEA-CH-3, IAEA-CH-6. IAEA-N-1 and IAEA-N-2, measurement error was estimated to be ±0.1 and ±0.2‰ for δ^13^C and δ^15^N, respectively. Only plasma values, which integrate isotopic information of <5 days [32] were used in the mixing model analysis.

Trophic enrichment factor values were calculated using linear models to estimate differences between sources (plasma and red cells) and prey (sardine) in R [34]. We determined proportion of each consumed prey by using mixing models in the R SIAR package [30]. The model was run using three basic matrices with stable isotope data from penguins (consumer), prey (source) and trophic enrichment factor (from this study). We then proceeded to run a posterior model adding external information (as in Jackson et al 2011), the so called priors. As the posterior model output will always be a combination of the prior and the maximum likelihood influence of the data, we used the matrix plot generated by SIAR [30] as a diagnostic measure to assist in detecting the influence of priors in the original model. Separated sources would provide more useful information for the data to override the prior. In contrast, when the model is struggling to separate sources, priors may have more influence in the posterior model.

Priors on prey proportion in the diet calculated from DNA analysis were determined, using published information of scats of individuals in the same feeding trials from this study [29]. The posterior model output was based on Dirichlet distribution which is used as prior distributions in Bayesian statistics [35].

### Ethics Statement

The project was approved by the Phillip Island Nature Park Animal Experimentation Ethics Committee (project 6.2007), with a research permit number 10004384 from the Department of Sustainability and Environment of Victoria, Australia. Captive adult penguins used in this project were from the Phillip Island Nature Parks rehabilitation centre, as part of a routine rehabilitation program, e.g. no birds were collected and kept in captivity for the solo purpose of this project.

## Results

### Trophic Enrichment Factor Value

There were significant differences between stable isotope values of fish muscle and penguin plasma and red cells with an exception of the carbon isotope values of fish muscle and red cells of penguins (Table 1). These estimated values for plasma were used in the further mixing model analysis.

**Table 1:** Trophic enrichment factor (TEF) values for carbon and nitrogen isotopes in plasma and red cells of little penguins.

Stable isotope	Blood tissue	TEF	SD	t value	p value
δ^15^ N	Plasma	4.05	0.95	17.48	<0.001
	Red cells	2.73	0.49	10.23	<0.001
δ^13^ C	Plasma	−1.91	1.49	−5.47	<0.001
	Red cells	−0.13	0.98	−0.33	0.74

The mean difference between prey and consumer values (TEF) in the linear model output are in comparison with stable isotope values of sardine muscle tissue. SD = standard deviation.

### Estimation of Diet from Mixing Models

**Figure 1:**
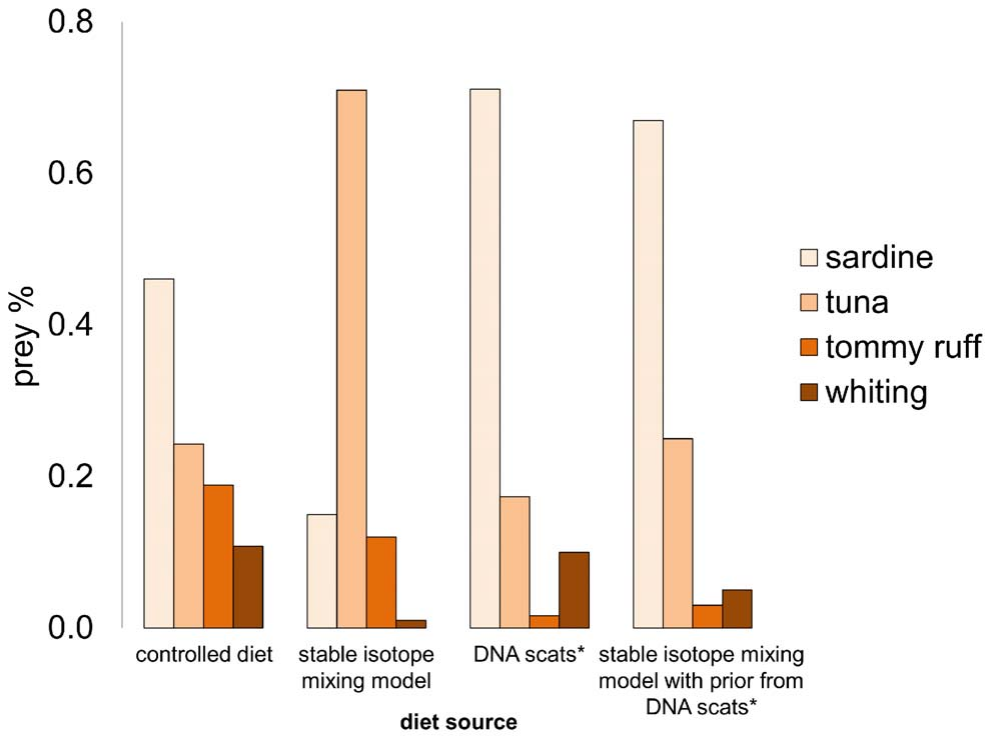
True proportion of prey types fed to penguins (controlled diet) and estimated proportion of these prey from different methods: mixing model [30], published information on DNA prey (*) from scats from the same experiment [29]. A posterior mixing model was run with stable isotope values using the DNA scats composition as priors [30].

**Figure 2:**
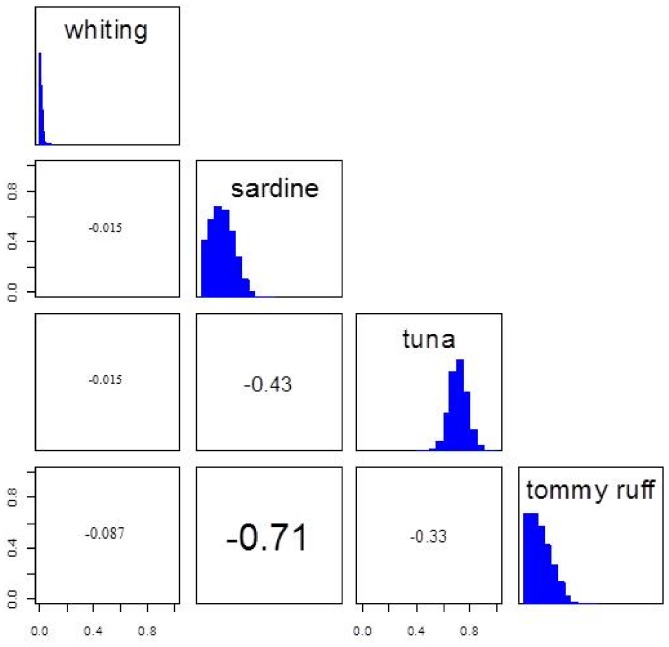
Matrix plot of estimate of each prey proportions calculated in the mixing models from the SIAR package output [30], represented by simulated values of the dietary proportions in the histograms (proportion in both axes). Correlation values between sources are inside the boxes to the left of histograms, with font size increasing from weak to strong correlation. Well separated sources resulted in weak correlation values (e.g. whiting vs. tuna, −0.015). Sources close to each other resulted in strong correlation (sardine vs. tommy ruff, −0.71). Increased correlation among sources will increase the level of uncertainty in the model output [37].

Penguins were fed a diet of whole defrosted sardine (46.1±8.2% of total dietary mass) complemented with a blended fish-shake diet which consisted of tuna (24.3±3.7%), tommy ruff (18.9±2.9%) and whiting (10.8±1.6%) (Fig. 1). We compared actual proportions of different prey types fed to penguins with the estimated proportions of these prey from mixing models based on the isotopic values of plasma of little penguins (Fig. 1 and 2). The two dominant prey taxa with highest proportion in the known diet exchanged positions in the mixing model results (Fig. 1). Sardine only accounted for 15% of the isotopic diet, while tuna accounted for 71% (Fig. 1). The contribution of the other two prey items differed to the actual dietary proportions, but tommy ruff’s contribution (19% to 12%) was higher than that of whiting (11% to 1%), in line with the actual controlled diet (Fig. 1).

Next, we ran a posterior mixing model adding priors, based on prey proportions obtained from the DNA analysis of scats from individuals in the same feeding trials from this study [29]. While the DNA proportion of prey in the scats predicted the position in terms of percentage of the two dominant prey, it did not match proportions by mass in the controlled diet (Fig. 1). The dominant food item in penguins’ scats was sardine, with a much higher proportion (71%) than the initial controlled diet (46%) [29].

When the mixing model was run with DNA priors, the posterior model output provided a distribution closer to the original controlled diet (Fig. 1). The distribution was clearly more influenced by the priors than the maximum likelihood influence of the data in the posterior model (Fig. 1). This is probably due to an increase in the level of uncertainty in the model caused by strong overlap among prey sources [36] given that sardine and tommy ruff had a strong negative correlation (−0.71, Fig. 2). However, when we summed each difference in prey proportion between the controlled diet and the other three methods used to determine prey composition, the posterior model (with priors) predicted the smallest difference (0.43) in the proportions of prey in relation to the controlled diet than the other two predicting proportions; mixing model (0.94) and DNA scats (0.50). These differences prey proportion among models are absolute values subtracted from the controlled diet.

## Discussion

We combined two independent sources of diet analysis to predict diet proportion from penguins fed a controlled diet. The output of the mixing model without priors showed a strong inversion in the main prey proportion in comparison with DNA analysis of penguin scats from same individuals under the same feeding experiment [29]. Results from the mixing model incorporating the DNA priory information (posterior model) suggested that the two techniques can be complementary by providing the closest output to the controlled (true) diet.

After the inclusion of priors, the mixing model resembled more closely the proportions provided by the prior information than the maximum likelihood solution informed by our mixing model data. The posterior model will always be a combination of the prior and the influence of the data [25], [35]. The less variation in the data and the more data there are, the more the posterior model will resemble the original data [25]. In contrast, the prior data can completely over-ride the original data. In case of stable isotope analysis, well separated isotopic signature of sources will provide more useful information for the data to over-ride the prior [26], [36]. In our stable isotope analysis, however, two source signatures (sardine and tommy ruff) were strongly correlated, which resulted in our posterior model having more influence of the prior. Normally, this would result in a high level of uncertainty in the model [37]. In our case, however, our priors were not an assessment of an experienced expert as advanced by Reverend Bayes [38] but qualified independent information of the diet from the same individuals. Indeed, the influence of prior information in the posterior distribution resulted in our best estimation of the true diet.

The results from the current feeding experiment produced a conflicting quantitative output. The dominant food item in the penguins’ controlled diet was sardine and this was the most common species recovered in the DNA analysis [29] but not in the stable isotope mixing model (Fig. 1). The reason for this inversion may be related to some degree of differences in prey assimilation efficiency (stable isotope) and digestion rate (DNA analysis). Mismatch between controlled (true) diet and dietary estimates from our isotope mixing model could be also the result of the so called “isotopic routing” [39], i.e. differential allocation of isotopically distinct macromolecules to consumer tissues. This is an unlikely explanation for this piscivorous species that feed exclusively on a protein-rich, fish diet [18].

In the controlled diet, the blended fish could have been assimilated more easily than whole sardines, and these blended fish were therefore over represented in the estimates from the stable isotope mixing model. In contrast, less assimilated/digested sardines appeared in higher proportion in the DNA scat analysis [29]. In nature, different prey species have different assimilation efficiency [40] so their dietary signatures would be incorporated into consumer tissues at different rates [24], [32], [33], [41] **.** By giving a controlled diet of whole and blended fish we have somewhat exaggerated differences in assimilation/digestion rates. Nevertheless, this artefact in our experiment can modulate the true diet in nature for animals, like little penguins, which feed on different sizes of prey [20]. Little penguins can feed on prey ranging from less than 1 cm (e.g. krill *Nyctiphanes australis* Sars, 1883) to up 30 cm (e.g. garfish *Hemiramphus far* Forsskål, 1775), [42] suggesting that differential digestion may occur. Further, diet segregation, particularly between parents and their chicks, are often determined by differences in isotopic signatures [8], [43], [44]. In the case of animals feeding their offspring with regurgitates, differences in stable isotope signatures can originate from difference in prey assimilation, given that parents feed their chicks highly digested fish [45] while parents feed on whole prey for themselves. Thus, our results on different estimates from stable isotope and DNA analysis highlight biases when trying to reconstruct the true diet consumed using techniques to examine assimilation (stable isotope) or undigested remains (DNA scats) diets.

Feeding experiments can reveal biases introduced by different methods of dietary studies. If the most important biases can be defined, then methods can be improved, or correction factors might be applied, to recover more accurate estimates to address specific questions on prey consumption. If it is required to determine which prey species are important to the consumer, measurements of the stable isotope ratios of carbon (δ^13^C) and nitrogen (δ^15^N) in mixing models can inform on prey assimilated by the predator [24]. If a study aims to determine a broad relative contribution of fish prey in the fed diets, DNA-based methods can provide useful information on identification of prey species and dietary diversity [17]. If a study requires the determination of consumed fish biomass for the management of fish stocks [1], [2], [46], the combined use of stable isotope and DNA-based technique in mixing model (this study) can provide more accurate information on the true diet consumed. Combining data from completely independent sources will also allow comparison between datasets and will moderate any systemic biases introduced through the different techniques [28], [47], [48]. The high taxonomic resolution provided by the DNA-based analysis of scat will also be useful for defining prey consumed in the wild on studies employing stable isotope analysis e.g. the field component of study [29]. While our posterior mixing model proportions of fish did not match the exact proportions by mass in the controlled diet, it has produced the best estimated model closer to the controlled diet proportion. Thus, our results are not the ultimate solution on the search for the true diet but these combined techniques will benefit further from current refinements in the mixing models [49] and diet assessment using next generation of DNA sequencing [17]. Since Deagle et al [29], more accurate DNA analysis have become available [50] and new stable isotope mixing models are fast evolving that could improve the currently problematic separation of two correlated source signatures [49]. Thus, our approach in this study can provide a powerful tool when searching for the true consumed diet by meso-top marine predators.
